# A ROS-responsive nanozyme delivering gallic acid synergistically ameliorates chronic rhinosinusitis via epithelial barrier restoration and Syk/NF-κB inhibition

**DOI:** 10.1016/j.ijpx.2026.100540

**Published:** 2026-04-11

**Authors:** Fangwei Zhou, Shanhu Gao, Tuotuo Xiong, Danyi Luo, Peixing Lin, Junbo Su, Houyong Kang

**Affiliations:** a Department of Otorhinolaryngology, The Second Affiliated Hospital of Chongqing Medical University, Chongqing 400010, China; b Department of Obstetrics and Gynecology, The Second Affiliated Hospital of Chongqing Medical University, Chongqing 400010, China

**Keywords:** Nanozyme, Gallic acid, Chronic rhinosinusitis, Epithelial barrier, ROS

## Abstract

**Background:**

Chronic rhinosinusitis (CRS) is a prevalent inflammatory disorder characterized by persistent mucosal inflammation and epithelial barrier dysfunction. However, effective therapeutic strategies targeting this core pathophysiology remain limited. Gallic acid (GA)—a polyphenol with known anti-inflammatory activity—suffers from poor bioavailability and the absence of targeted delivery, limiting its clinical translation.

**Methods:**

We engineered a reactive oxygen species (ROS)-responsive nanozyme, termed Ce-MOF-Pt@GA@PDA-TK-PEG (CP-GA-PKP), designed for targeted therapy of CRS. The material was characterized to confirm its structure and ROS-responsive properties. Its therapeutic efficacy was evaluated in a murine CRS model, with assessments focusing on inflammatory cell infiltration as well as the levels of ZO-1 and Occludin. Network pharmacology was employed to identify the molecular target of GA, which was subsequently validated through in vitro experiments using human nasal epithelial cells by analyzing the Syk/NF-κB pathway and downstream inflammatory cytokines.

**Results:**

In CRS mice, nanozyme treatment significantly alleviated nasal mucosal inflammation, reduced inflammatory cytokine levels, and restored Occludin and ZO-1 expression, indicating epithelial barrier repair. Network pharmacology identified Syk as the primary target of GA. Consistently, in vitro studies confirmed that the nanozyme inhibited the phosphorylation of Syk and its downstream effector NF-κB, thereby reducing pro-inflammatory cytokine levels.

**Conclusion:**

Our findings demonstrate that this ROS-responsive nanozyme provides a novel and effective therapeutic strategy for CRS in preclinical models, warranting further pharmacokinetic and toxicological evaluation to support clinical translation. It exerts synergistic therapeutic effects through a dual mechanism: by inhibiting the Syk/NF-κB signaling axis to suppress inflammation, and by protecting epithelial barrier integrity. A promising nanotherapeutic strategy is proposed and the molecular mechanism underlying GA's action in CRS is investigated.

## Graphical abstract

Scheme 1. Schematic illustration of ROS-responsive CP-GA-PKP nanozyme in the inflammatory microenvironment of CRS and the corresponding mechanism of enhancing anti-inflammatory and epithelial barrier protection.

**Figure:**
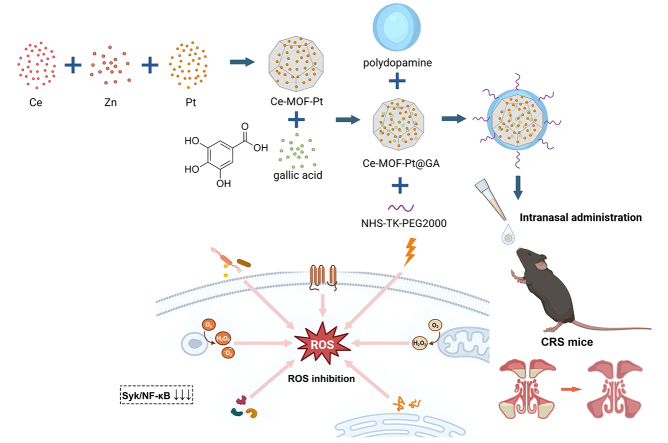

## 1 Introduction

Chronic rhinosinusitis (CRS), an inflammatory disorder of the nasal and paranasal sinus mucosa persisting >12 weeks and resistant to standard care, poses a major public-health burden (Fokkens et al., 2020). While the pathophysiology of CRS is undeniably complex, involving dysbiosis, immune dysregulation, and biofilm formation, a growing consensus indicates that the breakdown of the sinonasal epithelial barrier is a critical initiating and perpetuating factor (Jiao et al., 2019). This tightly regulated physical and functional barrier, composed of epithelial cells interconnected by tight junction proteins such as Zonula Occludens-1 (ZO-1) and Occludin, serves as the first line of defense against inhaled pathogens, allergens, and environmental irritants (Soyka et al., 2012). Its compromise, evidenced by the downregulation of tight junction proteins and enhanced mucosal permeability, allows for the uncontrolled translocation of antigens and microbes, thereby triggering a robust and sustained immune response that fuels the chronic inflammatory cycle characteristic of CRS (Huang et al., 2024).

Among the factors driving epithelial barrier disruption, the overproduction of reactive oxygen species (ROS) in the sinonasal microenvironment constitutes a central driver (Kojima et al., 2013). Inflammatory cells like neutrophils and eosinophils generate excessive ROS as a host defense mechanism, but chronic overproduction leads to oxidative stress. This state damages epithelial cells through lipid peroxidation, protein dysfunction, and DNA damage (Luu Quoc et al., 2021) and directly contributes to tight junction disassembly, worsening barrier hyperpermeability and creating a vicious cycle of inflammation and tissue damage (Comstock et al., 2011). Therefore, a therapeutic strategy that directly targets the oxidative microenvironment to simultaneously scavenge excess ROS and promote epithelial barrier repair represents a promising, mechanism-driven approach for CRS. Current first-line treatments, such as intranasal corticosteroids and saline irrigations, primarily suppress inflammation but often provide incomplete relief and fail to directly correct barrier dysfunction (De Corso et al., 2022). Furthermore, thick mucus and bacterial biofilms in CRS can impede drug penetration, limiting treatment efficacy (Maina et al., 2018). This underscores the clear need for novel therapeutic platforms that enable targeted delivery to the sinus mucosa and can disrupt the pathogenic cycle of oxidative stress and barrier impairment. Consequently, an ideal therapy for CRS would not only possess potent anti-inflammatory and antioxidant properties but also directly reinforce the epithelial barrier and achieve targeted accumulation in the inflamed sinuses.

To address these challenges, we turned to nanotechnology. We developed a multi-functional platform based on cerium-based metal-organic frameworks (Ce-MOFs), a class of nanozymes with enzyme-mimetic activities (Huang et al., 2019). Ce-MOFs exhibit dynamic redox properties, enabling them to mimic both catalase and superoxide dismutase (SOD), thereby efficiently scavenging pathological ROS in CRS (Liu et al., 2022). By decomposing H₂O₂, they not only alleviate oxidative stress but also generate oxygen, potentially mitigating tissue hypoxia (Alizadeh et al., 2020). The incorporation of platinum nanoparticles (Pt) further enhances their antioxidant capacity, forming a synergistic nanozyme system for efficient ROS elimination (Moglianetti et al., 2016). To simultaneously promote barrier repair, we incorporated gallic acid (GA), a natural polyphenol known for its ability to upregulate tight junction proteins and restore epithelial integrity (Arinno et al., 2025; Bhuia et al., 2023; Chu et al., 2024). Nanocarrier encapsulation of GA improves its bioavailability and enables sustained release at the damaged barrier site.

For targeted delivery, the system is coated with a polydopamine (PDA) layer, which enhances mucosal adhesion and provides additional antioxidant activity (Liu et al., 2014). A key innovation is the incorporation of a thioketal (TK) linker between the PDA coating and the outer PEG layer. Thioketal bonds are specifically cleaved by ROS, particularly H₂O₂, functioning as a “ROS-responsive gatekeeper” (Shim and Xia, 2013). In the oxidative CRS microenvironment, TK cleavage removes the PEG layer, exposing the adhesive PDA surface and triggering rapid drug release (Yang et al., 2021). This ROS-responsive mechanism not only enhances cellular uptake but also is designed to enable targeted delivery to sites of high ROS concentration and epithelial damage (Lee et al., 2021).

Herein, we propose the rational design and synthesis of a novel, multi-functional nanotherapeutic platform: Ce-MOF-Pt@GA@PDA-TK-PEG (CP-GA-PKP). This system is engineered to actively target the core pathological mechanisms of CRS, with a primary focus on restoring the sinonasal epithelial barrier. We hypothesize that upon intranasal administration, CP-GA-PKP will: (1) accumulate in the inflamed sinus mucosa; (2) respond to the high H₂O₂ levels via its TK linker, enhancing cellular internalization; (3) potently scavenge excess ROS through the synergistic action of the Ce-MOF-Pt nanozyme core and the PDA shell, thereby mitigating oxidative stress; and (4) release GA to directly promote the reinstatement of tight junction integrity and barrier function. By concurrently breaking the cycle of oxidative damage and actively facilitating barrier repair, the CP-GA-PKP platform represents a paradigm shift in CRS therapy, moving beyond symptomatic control towards targeted pathophysiological restoration. This work aims to comprehensively evaluate this hypothesis through in vitro and in vivo experiments, providing preclinical validation for this innovative strategy and a promising therapeutic candidate for recalcitrant CRS.

## 2 Materials and methods

### 2.1 Synthesis and characterization of nanoparticle

#### 2.1.1 Synthesis of Ce-MOF-Pt

Dissolving 0.77 g of 2-methylimidazole in 2.655 mL of methanol yielded a solution. Under continuous stirring, Zn(NO₃)₂·6H₂O and Ce(NO₃)₃·6H₂O were introduced into the solution, maintaining a Ce:Zn molar ratio of 1:50. The solid product was isolated by first stirring the mixture at room temperature for 30 min, followed by centrifugation at 8000 rpm for 10 min. The resulting precipitate underwent multiple rinses with deionized water to yield the Ce-MOF precursor. Next, H₂PtCl₆ was mixed with the obtained Ce-MOF at a mass ratio of 0.2:5 under vigorous stirring for 1 h. Subsequently, 2 mL of a freshly prepared NaBH₄ solution (2 mg/mL) was added, and vigorous stirring was continued for an additional 3 h. The product was isolated via centrifugation (10,000 rpm, 15 min), subjected to three rounds of washing with deionized water, and ultimately re-dispersed in deionized water, yielding the final product Ce-MOF-Pt.

#### 2.1.2 Synthesis of Ce-MOF-Pt@GA

GA was loaded onto the pre-synthesized Ce-MOF-Pt nanoparticles via an adsorption method. The as-synthesized Ce-MOF-Pt nanoparticles (50 mg) were added to 20 mL of an aqueous GA solution (concentration: 2 mg/mL). The GA solution was adjusted to a pH of 5.0 using 0.1 M NaOH. This condition enhances GA's solubility and facilitates its interaction with the nanoparticle surface through hydrogen bonding or deprotonation. Under continuous stirring in the dark at room temperature (25 °C) for 24 h, adsorption equilibrium was ensured. Subsequently, the product (Ce-MOF-Pt@GA) was collected by centrifugation at 10,000 rpm for 15 min. The precipitate was rinsed three times with deionized water (pH 5.0, adjusted with dilute HCl) to remove unbound GA while minimizing premature desorption. Finally, the nanoparticles were re-dispersed in 10 mL of deionized water for subsequent applications.

#### 2.1.3 Synthesis of CP-GA-PKP

First, Ce-MOF-Pt@GA was dispersed in ethanol, to which an ammonia aqueous solution was added under stirring for 30 min. Following the introduction of dopamine hydrochloride, the reaction was continued to proceed for 6 h. The resultant particles, denoted as Ce-MOF-Pt@GA@PDA, were harvested via centrifugation and then underwent three rounds of rinsing with deionized water. Subsequently, the as-synthesized Ce-MOF-Pt@GA@PDA was suspended in HEPES buffer and added dropwise into a solution of pre-dissolved NHS-TK-PEG2000. The reaction mixture was stirred vigorously for 4 h. Following centrifugation at 12,000 rpm for 15 min, the final material was harvested, rinsed thrice with deionized water, and obtained as the target materials, denoted Ce-MOF-Pt@GA@PDA-TK-PEG2000 (CP-GA-PKP).

#### 2.1.4 Characterization of Nanoparticles

The hydrodynamic size and zeta potential of the nanoparticles were measured using a Brookhaven NanoBrook 90Plus PALS analyzer (USA). Morphological and elemental analyses were performed with transmission electron microscopy (TEM) and energy-dispersive X-ray spectroscopy (EDS) mapping on a Thermo Scientific TF20 microscope (USA), as well as scanning electron microscopy (SEM) on a ZEISS Sigma 300 field-emission SEM (Germany). The crystallinity was assessed by X-ray diffraction (XRD) using a Bruker D8 Advance diffractometer (Germany). Surface chemical states were analyzed via X-ray photoelectron spectroscopy (XPS) on a Thermo Scientific K-Alpha spectrometer (USA), and functional groups were characterized by Fourier transform infrared (FTIR) spectroscopy using a Shimadzu IRSpirit-X series spectrophotometer (Japan). The encapsulation efficiency (EE) and drug loading capacity (DL) of GA were also assessed. After synthesis, free GA in the supernatant was separated by centrifugation and quantified via high-performance liquid chromatography (HPLC). EE and DL were respectively calculated as the ratio of encapsulated GA to the total amount added, and the ratio of encapsulated GA weight to the total weight of dried nanoparticles.

#### 2.1.5 In vitro ROS-responsive drug release profile

The ROS-triggered release of GA from CP-GA-PKP was evaluated in phosphate-buffered saline (PBS) with or without 10 mM H₂O₂. Dispersed in 10 mL of release medium, CP-GA-PKP nanoparticles (containing 1 mg GA) were maintained at 37 °C with gentle shaking (100 rpm). At predetermined intervals (0.5, 1, 2, 4, 8, 12, and 24 h), 1 mL aliquots were collected, subjected to centrifugation (12,000 rpm, 10 min), and analyzed by HPLC to quantify supernatant GA concentration. The cumulative release percentage was then calculated and plotted versus time.

#### 2.1.6 SOD-like activity assay

The SOD-like activity of Ce-MOF-Pt was evaluated using a WST-1-based Superoxide Dismutase Assay Kit (Dojindo, Japan) according to the manufacturer's instructions. Briefly, different concentrations of Ce-MOF-Pt nanoparticles were added to a 96-well plate containing WST-1 working solution. The reaction was initiated by adding xanthine oxidase, and the mixture was incubated at 37 °C for 20 min. The absorbance at 450 nm was measured using a microplate reader. The inhibition rate was calculated as follows: Inhibition rate (%) = [(Ablank - Asample)/Ablank] × 100%, where Ablank is the absorbance of the control group without nanoparticles. The concentration required to achieve 50% inhibition (IC50) was calculated, and the SOD-like activity was expressed as U/mg (1 U is defined as the amount of nanozyme that inhibits the reduction of WST-1 by 50%).

### 2.2 Network pharmacology analysis

#### 2.2.1 Collection of the targets

The canonical SMILES format of GA was downloaded from PubChem (https://pubchem.ncbi.nlm.nih.gov/). Potential GA targets were retrieved from three widely used databases: SwissTargetPrediction (based on ligand similarity), ChEMBL (based on bioactivity data), and TargetNet (based on structure-based prediction). This combination of ligand-based and structure-based approaches ensures comprehensive coverage of potential GA targets (Daina et al., 2019; Zdrazil et al., 2024; Yao et al., 2016). While CRS-associated targets were acquired from GeneCards (https://www.genecards.org/) and OMIM (https://omim.org/) using “chronic rhinosinusitis” as the search term. After merging the collected targets and removing duplicates, the intersection between the GA-predicted and CRS-related target sets was defined as potential therapeutic targets for subsequent analysis.

#### 2.2.2 Protein-protein interaction (PPI) network construction

The overlapping targets were employed to construct a PPI network using the STRING platform. For visualization and hub target identification, Cytoscape 3.7.2 was applied, with hub identification based on scores of MCC, Degree, and Closeness centrality.

#### 2.2.3 Gene Ontology (GO) and Kyoto Encyclopedia of Genes and Genomes (KEGG) pathway enrichment

To clarify the biological roles and signal transduction pathways linked to the intersecting targets, we conducted GO and KEGG pathway enrichment analyses with the “clusterProfiler” R package (version 4.10.0). A *P* value <0.05 served as the significance criterion for the functional enrichment.

### 2.3 Differential Gene Expression (DEGs) analysis

The GSE136825 dataset contains transcriptomic profiles of 28 control and 42 CRS samples. DEGs were detected by the “limma” R package (v3.58.1). DEGs were screened using thresholds of a P value <0.05 and |log2 FC| > 0.585. Subsequently, their expression patterns were visualized via volcano plots and heatmaps, created with the “ggplot2” (v3.5.1) and “pheatmap” (v1.0.12) R packages, respectively.

### 2.4 Molecular docking

Molecular docking between GA and the identified hub targets was conducted with the CB-Dock2 server (https://cadd.labshare.cn/cb-dock2/php/index.php), a computational tool built on AutoDock Vina that enables automated detection and assessment of ligand-receptor binding interactions. The two-dimensional structural formula of GA in SDF format was retrieved from the PubChem repository and uploaded to CB-Dock2. From the RCSB PDB (https://www.rcsb.org/), the corresponding crystal structures of the target proteins were acquired and then imported into the platform. Prior to docking, all protein structures underwent preparation, which involved hydrogen atom addition and water molecule removal. CB-Dock2 was used to compute the binding affinity (expressed in kcal/mol), and the pose with the most favorable docking score was selected as the optimal model.

### 2.5 Animals

SPF-grade male C57BL/6 mice (age, 6–8 weeks; weight, 18–25 g) were purchased from the Experimental Animal Centre of Chongqing Medical University. Mice were housed in a specific-pathogen-free environment at standard temperature (18–22 °C), moderate humidity (50–55%), and under a 12 h light/dark cycle. All mice were acclimated in the animal room for 1 week before being involved in experimental procedures. The animal experimental protocols were approved by the Ethics Committee of Chongqing Medical University Animal Centre (IACUC-SAHCQMU-2025-0114).

### 2.6 CRS modeling, grouping, and intervention

Mice that died during the experimental procedure or showed signs of severe distress (e.g., >20% body weight loss, inability to eat or drink) were excluded from the final analysis. No animals met the exclusion criteria in this study. We randomly assigned the mice into five groups (*n* = 10 per group): Control (Ctrl), CRS, CRS + CP-PKP, CRS + GA, and CRS + CP-GA-PKP, and established a mouse CRS model following a previously reported method (Kim et al., 2011). On days 0 and 5, experimental group animals were intraperitoneally (i.p.) sensitized with ovalbumin (25 mg, OVA, Sigma-Aldrich) in 200 μL of PBS containing aluminum hydroxide (2 mg). This was followed by continuous intranasal (i.n.) challenges with 20 μL of a 3% OVA solution over a period of 13 weeks. For the final 8 weeks, the animals were additionally challenged intranasally with both staphylococcal enterotoxin B (10 ng SEB, Toxin Technology, USA) and OVA. Throughout the experimental period, the control (Ctrl) group was administered PBS as a comparative baseline. Following the completion of the 13-week modeling protocol, the CRS + CP-PKP, CRS + GA, and CRS + CP-GA-PKP groups received an additional week of therapeutic intervention. During this period, mice were administered daily intranasal doses equivalent to 50 μg of gallic acid. The CRS + GA group received 50 μg of free GA dissolved in PBS. The CRS + CP-GA-PKP group received 0.436 mg of CP-GA-PKP (containing 50 μg GA, based on a drug loading of 11.45%) dissolved in PBS. The CRS + CP-PKP group received 0.436 mg of the empty nanocarrier as a vehicle control. After the completion of treatments and euthanasia, the blood samples were drawn for biochemistry and routine blood examination. Major organs (heart, liver, spleen, lungs, and kidneys) were harvested for H&E staining.

### 2.7 H&E and periodic acid-Schiff (PAS) staining

Nasal mucosal tissues and major organs from mice were immersed in 4% paraformaldehyde for 48 h, subjected to decalcification in ethylenediaminetetraacetic acid for 1 week, and processed for embedding in paraffin. The blocks were sectioned at 4 μm. Nasal mucosal sections were processed for HE and PAS staining, whereas the histological changes of organs were also evaluated using HE staining. All stained sections were assessed and imaged using a microscope. In three randomly chosen high-power fields (400×), eosinophils and goblet cells were quantified using Image-Pro Plus v6.0 and Image J software. All histological evaluations and quantifications (eosinophils, goblet cells) were performed by two independent investigators who were blinded to the group allocation.

### 2.8 Quantitative RT-PCR analysis

Total RNA was isolated from homogenized nasal mucosa (collected per group) via a column-based method, with purity and concentration verified by UV spectrophotometry. Subsequently, the RNA was reverse transcribed to generate cDNA for quantitative PCR (qPCR) using the SYBR Green method. Real-time monitoring of PCR product accumulation allowed for the recording of cycle threshold (Ct) values, and the 2-ΔΔCt method was applied to evaluate gene expression levels. All primers were synthesized by Sangon Biotech Co., Ltd. The mouse primer sequences were as follows: GAPDH – forward: AGGTCGGTGTGAACGGATTTG, reverse: TGTAGACCATGTAGTTGAGGTCA; Syk – forward: CTTCTGGCTCACAAGGACATGG, reverse: GGAAACATGATTAAGTCGCCGTG; TP53 – forward: AGACCGCCGTACAGAAGAAGAAA, reverse: CGGAACATCTCGAAGCGTTTAC.

### 2.9 Cell culture and treatment

Human nasal epithelial cells (HNEpCs) were purchased from Wuhan Procell Biotechnology Co., Ltd. In RPMI 1640 medium (Gibco) supplemented with 10% fetal bovine serum (Gibco) and 1% penicillin-streptomycin (Sigma-Aldrich), cells were cultured and maintained at 37 °C in a humidified atmosphere of 5% CO₂. The experiment comprised five cellular groups: Ctrl, LPS, LPS + GA, LPS + CP-GA-PKP, and LPS + PBS. Prior to treatment, cells in the LPS-stimulated groups were pretreated with or without the respective compounds for 1 h, and then stimulated with lipopolysaccharide (LPS, 1 μg/mL) for 24 h to mimic an inflammatory microenvironment. The intervention groups received the following treatments: LPS + GA was co-treated with GA at a final concentration of 10 μM (1.7 μg/mL); LPS + CP-GA-PKP was co-treated with CP-GA-PKP at a final concentration of 14.85 μg/mL (containing an equivalent amount of GA, 1.7 μg/mL, based on a drug loading of 11.45%); and the LPS + PBS group served as the vehicle control, receiving an equal volume of PBS. The Ctrl group was cultured in complete medium without LPS or compound treatment.

### 2.10 Transepithelial electrical resistance (TEER) measurement

To functionally assess epithelial barrier integrity, HNEpCs were seeded on Transwell inserts (0.4 μm pore size, Corning, USA) at a density of 1 × 10^5^ cells per insert and cultured until confluence. Cells were then subjected to the same treatments as described in Section 2.9: Ctrl, LPS, LPS + GA, LPS + CP-GA-PKP, and LPS + PBS. TEER values were measured at 0, 6, 12, and 24 h post-LPS stimulation using an epithelial volt-ohmmeter (EVOM2, World Precision Instruments, USA). The measured resistance values were multiplied by the membrane area to obtain Ω·cm^2^, and results were expressed as percentage of baseline values (time 0).

### 2.11 Immunofluorescence

Immunohistochemical staining was conducted to visualize the localization and abundance of ZO-1 and Occludin proteins in both nasal mucosal and cultured cells. Paraffin-embedded tissue sections underwent dewaxing and antigen retrieval. Cultured cell monolayers were rinsed with cold PBS, fixed using 4% paraformaldehyde (PFA) for 30 min, and then permeabilized with 0.2% Triton X-100. All samples were blocked with 10% goat serum (1 h, room temperature) prior to overnight incubation at 4 °C with anti-ZO-1 (1:100, Abcam) and anti-Occludin (1:100, Abcam) primary antibodies. After washing, samples were incubated with corresponding fluorophore-conjugated secondary antibodies at room temperature for 2 h. Nuclei were counterstained with DAPI. Images were acquired with an Olympus fluorescence microscope. Quantitative assessment was performed by analyzing three randomly selected high-power fields from each tissue section.

### 2.12 Detection of malondialdehyde (MDA) and SOD levels in nasal mucosa

Nasal mucosa tissues were homogenized on ice in nine volumes of cold 0.9% saline (weight-to-volume ratio of 1:9) to obtain a 10% homogenate. After centrifugation (3000 r/min, 10 min, 4 °C), the supernatant was collected for further analysis. The protein concentration of the supernatant was measured to normalize subsequent data. Following the manufacturers' protocols, MDA levels and SOD activity in the supernatant were assessed via specific commercial assay kits. Thus, MDA (a marker of lipid peroxidation) and SOD (a critical antioxidant enzyme) were measured to investigate the oxidative stress status in the nasal mucosa across all experimental groups.

### 2.13 Western blotting

Total protein was isolated from cells and nasal mucosal tissues via RIPA lysis buffer (Servicebio, China). Protein concentration was determined with a BCA assay kit (Servicebio, China). Equal amounts of protein samples (40 μg per lane) were separated by 10% SDS-PAGE and then transferred onto polyvinylidene difluoride (PVDF) membranes. The membranes were blocked and subsequently incubated overnight at 4 °C with primary antibodies against the following proteins: Syk (1:1000; Abcam), IκBα (1:1000; Abcam), p65 (1:1000; Abcam), phospho-p65 (p-p65; 1:1000; Abcam), TP53 (1:1000; Abcam), Occludin (1:1000; Abcam), ZO-1 (1:1000; Abcam), and GAPDH (1:500; Abcam). Following three washes with TBST (Tris-buffered saline containing 0.1% Tween-20), the membranes were incubated with an HRP-conjugated goat anti-rabbit secondary antibody (1:3000; Abcam) at room temperature for 1 h, followed by protein band visualization using a Servicebio® enhanced chemiluminescence (ECL) substrate kit (Wuhan Servicebio Technology Co., Ltd.). GAPDH served as the loading control. Semi-quantification of band intensity was performed using ImageJ software (version 1.52a; NIH, USA).

### 2.14 Measurement of Inflammatory Cytokines by ELISA

Cytokine levels in nasal lavage fluid (NLF) were quantified as follows: After anesthesia, mice were subjected to tracheotomy. A catheter was then inserted through the upper airway into the nasopharynx to gently infuse 1 mL of chilled PBS, following which NLF was collected from the nostrils. The concentrations of interleukin (IL)-4, IL-5, IL-13, and interferon (IFN)-γ in NLF were quantified using commercial mouse-specific ELISA kits (R&D Systems), strictly according to the manufacturer's instructions.

In cell culture supernatants, the concentrations of IL-1β, IL-6, and tumor necrosis factor (TNF)-α were quantified using the same type of mouse-specific ELISA kits (R&D Systems), in accordance with the manufacturer's protocols.

### 2.15 Statistical analysis

All experimental data are presented as the mean ± standard deviation (SD). Statistical differences between two groups were assessed with Student's *t*-test. For comparisons among more than two groups, one-way analysis of variance was performed, followed by Bonferroni's post hoc test for multiple comparisons. A *P*-value of less than 0.05 was considered statistically significant. All statistical analyses were performed using GraphPad Prism software (version 8.0; GraphPad Software, USA).

## 3 Result

### 3.1 Synthesis and Characterization of Nanoparticles

**Fig. 1:**
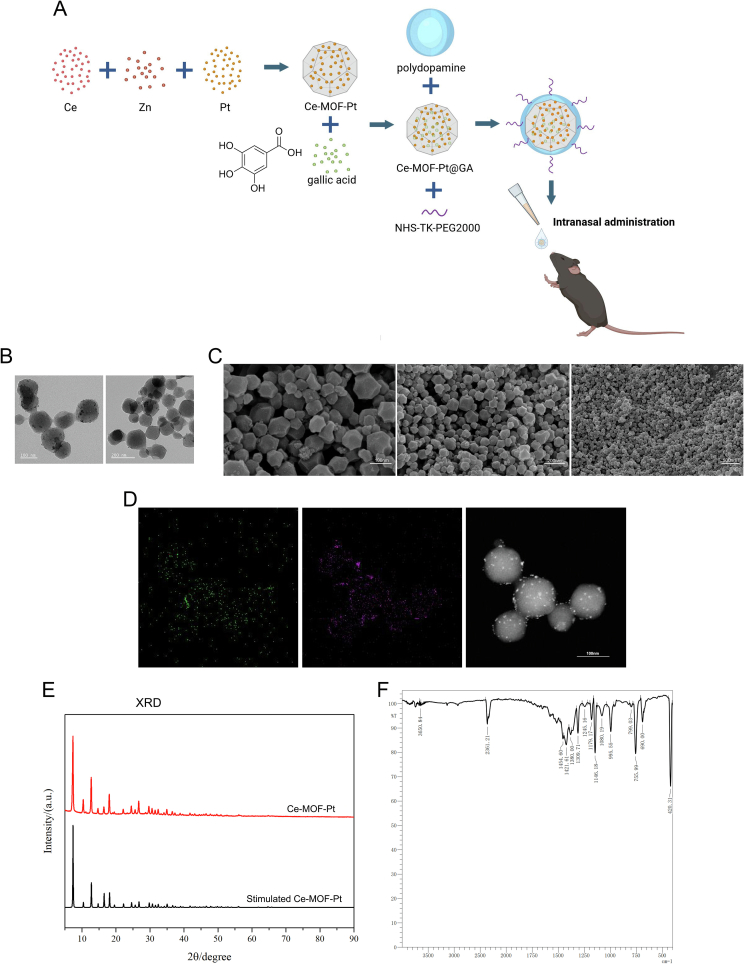
Characterizations of Ce-MOF-Pt. (A) Schematic illustration of the preparation of CP-GA-PKP and its collaborative therapy for CRS. (B) TEM image. (C) SEM image. (D) Elemental mapping. (E) XRD patterns of Ce-MOF-Pt and simulated patterns. (F) FT-IR spectra.

The multifunctional nanoplatform Ce-MOF-Pt@GA@PDA-TK-PEG (CP-GA-PKP) was constructed through a sequential synthesis strategy, as illustrated in Fig. 1A. First, the Ce-MOF-Pt (CP) nanozyme core was synthesized. TEM and SEM images confirmed that the CP nanoparticles possessed a uniform rhombic dodecahedron morphology, characteristic of the ZIF-8 template (Fig. 1B-C). Elemental mapping verified the homogeneous distribution of cerium (Ce) and platinum (Pt) within the framework, indicating successful doping (Fig. 1D). XRD analysis indicated that the diffraction peaks of CP closely matched the simulated pattern of ZIF-8 (particularly at 2θ ≈ 7.3°, 10.4°, 12.7°, 14.7°, 16.4°, and 18.0°), confirming its well-defined crystalline structure with sodalite topology (Fig. 1E). Fourier transform infrared (FT-IR) spectroscopy further identified key functional groups: absorption peaks at 3130 cm^−1^ and 2930 cm^−1^ (C—H stretching), 1580 cm^−1^ (C=N stretching), and in the range of 420–760 cm^−1^ (Zn—N vibrations), collectively verifying the intact ZIF-8 framework (Fig. 1F).

**Fig. 2:**
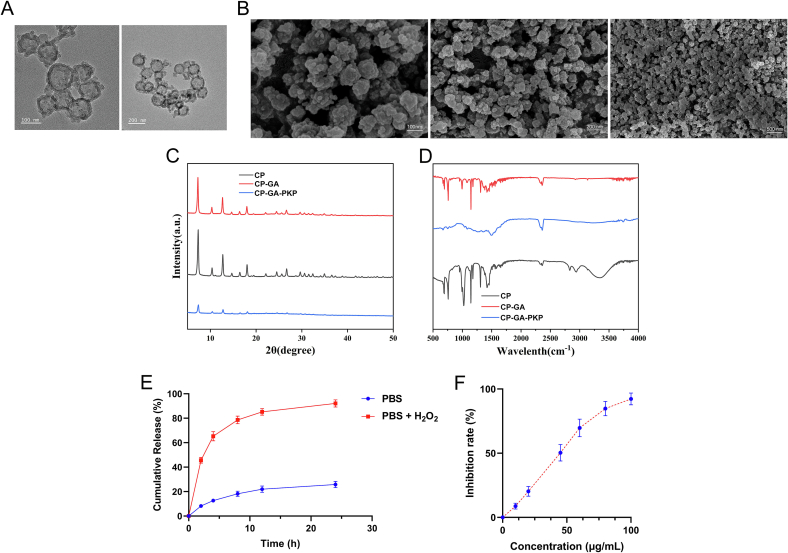
Characterizations of CP-GA-PKP. (A) TEM image. (B) SEM image. (C) XRD patterns of CP, CP-GA, and CP-GA-PKP. (D) FT-IR spectra of CP, CP-GA, and CP-GA-PKP. (E) ROS-responsive drug release profiles of CP-GA-PKP. Cumulative release of GA from CP-GA-PKP in PBS (pH 7.4) without (blue) or with (red) 10 mM H₂O₂ at 37 °C. (F) SOD-like activity of Ce-MOF-Pt measured by WST-1 assay. The IC50 value was calculated to be 45.3 ± 4.5 μg/mL. Data are presented as mean ± SD (*n* = 3). (For interpretation of the references to colour in this figure legend, the reader is referred to the web version of this article.)

Subsequently, the CP core was functionalized through GA loading, polydopamine (PDA) coating, and thioketal-polyethylene glycol (TK-PEG) conjugation to obtain the final CP-GA-PKP nanocomposite. TEM and SEM images revealed that the spherical CP-GA-PKP nanoparticles were larger than the polyhedral CP core, visually confirming the successful surface modifications (Fig. 2A-B). XRD patterns demonstrated that the crystalline framework remained intact throughout these steps, as indicated by peak positions matching those of pristine CP and standard ZIF-8 (Fig. 2C). FT-IR spectroscopy provided chemical evidence for each modification step: the spectrum of CP@GA showed a broad O—H stretching band (∼3350 cm^−1^) and a C=O stretching vibration (∼1700 cm^−1^) characteristic of GA, while the spectrum of CP-GA-PKP displayed new peaks at ∼1600 cm^−1^ (aromatic C=C/N–H bending), ∼1500 cm^−1^ (aromatic C=C stretch), and ∼ 1250 cm^−1^ (C–O–C/C–N vibrations), confirming the presence of the PDA coating (Fig. 2D).

The physicochemical properties of the nanoparticles at each stage were quantified. Dynamic light scattering measurements revealed that the hydrodynamic diameter increased from 159.2 ± 8.0 nm for CP to 234.8 ± 18.7 nm for CP@GA, and finally to 390.6 ± 22.0 nm for CP-GA-PKP, with corresponding polydispersity indices (PDI) of 0.11 ± 0.02, 0.15 ± 0.03, and 0.18 ± 0.02, respectively, indicating good colloidal stability. The zeta potential shifted from slightly positive (+3.13 ± 0.14 mV for CP) to negative after GA loading (−17.1 ± 1.06 mV for CP@GA), and remained negative after further modifications (−12.82 ± 1.23 mV for CP-GA-PKP). HPLC analysis determined the EE and DL of GA in CP-GA-PKP. All measurements were performed in triplicate, and the results were expressed as mean ± standard deviation. The EE was 91.66% ± 1.28% (*n* = 3), and the DL was 11.45% ± 0.49% (n = 3). HPLC conditions for GA quantification were as follows: chromatographic separation was performed on a Waters Symmetry C18 column (5 μm, 4.6 mm × 150 mm). The mobile phase consisted of methanol - 0.05% phosphoric acid solution at a flow rate of 0.8 mL/min. The detection wavelength was set at 273 nm, and the injection volume was 20 μL. The method was validated for linearity (R^2^ > 0.999), and the average recovery rate was 98.50% ± 1.06% (n = 3). The stepwise increase in hydrodynamic diameter, along with the corresponding changes in zeta potential (from positive to negative after GA loading), confirmed the successful loading of GA and the subsequent coating with PDA and PEG.

To validate the ROS-responsive property of CP-GA-PKP, drug release profiles were examined under oxidative conditions mimicking the CRS microenvironment. As shown in Fig. 2E, in PBS alone, CP-GA-PKP exhibited sustained release with less than 25% of GA released within 24 h. In stark contrast, in the presence of 10 mM H₂O₂, over 85% of encapsulated GA was rapidly released within 12 h. This pronounced acceleration confirmed the thioketal linker-mediated, ROS-triggered drug release behavior, ensuring on-demand GA delivery at inflamed sites with elevated ROS levels.

To quantitatively evaluate the antioxidant capacity of the nanozyme core, we assessed the SOD-like activity of Ce-MOF-Pt using a WST-1 assay. As shown in Fig. 2F, Ce-MOF-Pt scavenged superoxide anions in a concentration-dependent manner. The IC50 value was calculated to be 45.3 ± 4.5 μg/mL (*n* = 3). Based on the manufacturer's definition (one unit U = amount inhibiting WST-1 reduction by 50% in the 200 μL reaction system), the specific activity was calculated to be 22.1 ± 2.2 U/mg. This result provides direct evidence that the Ce-MOF-Pt core possesses intrinsic SOD-mimicking enzymatic activity, contributing to the overall ROS-scavenging capacity of the CP-GA-PKP platform.

### 3.2 Intersection targets of GA and CRS

**Fig. 3:**
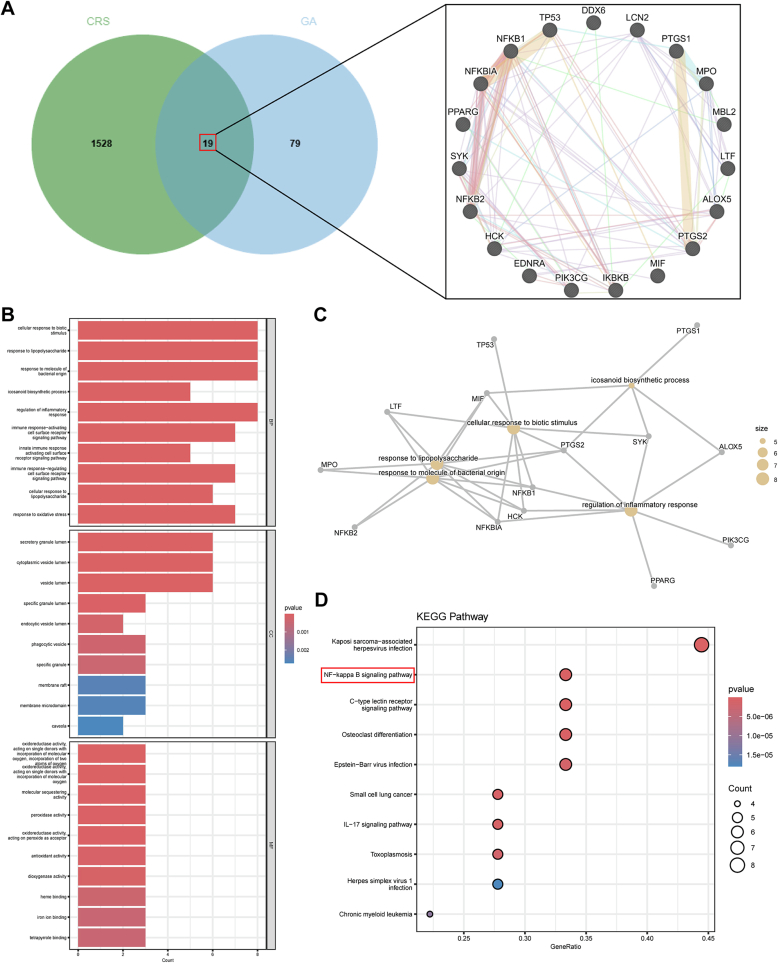
Bioinformatic analysis of the common targets between GA and CRS. (A) Venn diagram showing the overlapping targets of GA and CRS. (B) GO enrichment analysis of the common targets. (C) CNet plot visualizing the GO enrichment results. (D) KEGG pathway enrichment analysis of the common targets.

Screening of the Swiss Target Prediction and ChEMBL databases yielded 98 unique GA-related targets. Separately, 1547 CRS-associated targets were retrieved from the GeneCards and OMIM databases. As shown in Fig. 3A, a total of 19 overlapping targets were identified by Venn diagram analysis.

### 3.3 GO and KEGG pathway enrichment analysis

As shown in Fig. 3B, GO analysis was performed, identifying 10 entries each for biological processes, cellular components, and molecular functions, such as regulation of inflammatory response, response to oxidative stress, and antioxidant activity, among others. To visually demonstrate the functional relationships among the enriched GO terms, we constructed a CNET plot. As shown in the Fig. 3C, the enriched terms are primarily clustered into several core functional modules, such as “regulation of inflammatory response “, “cellular response to biotic stimulus”, and “response to molecule of bacterial origin”. KEGG enrichment analysis was subsequently performed to investigate the signaling pathways underlying GA's therapeutic effect on CRS. This analysis highlighted the top 10 pathways, notably encompassing NF-κB and IL-17 signaling pathways (Fig. 3D).

### 3.4 Identification of critical targets for GA in the treatment of CRS via GEO dataset analysis

**Fig. 4:**
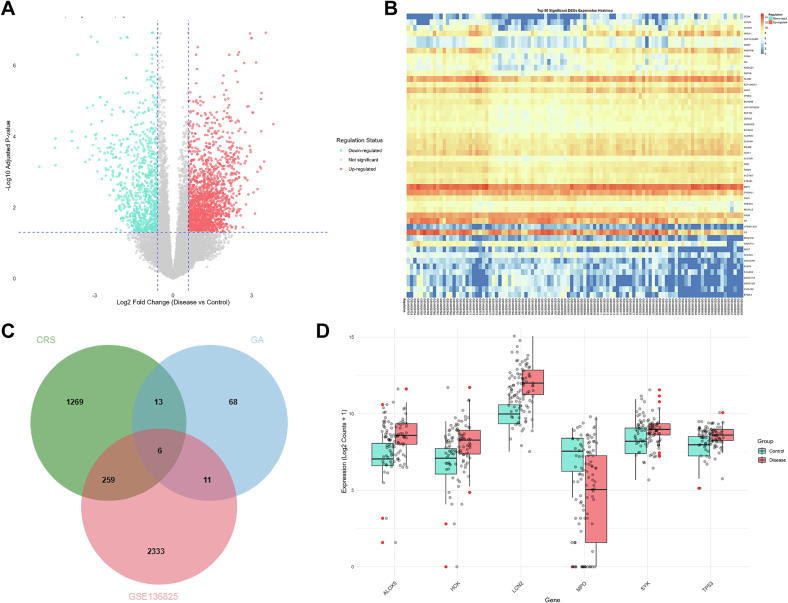
Identification of critical targets for GA in treating CRS through analysis of GEO datasets. (A) Volcano plot of differentially expressed genes (DEGs) in the CRS dataset GSE136825. (B) Heatmap of DEGs in the CRS dataset GSE136825. (C) Venn diagram identifying the common targets among GA, CRS, and the DEGs from GSE136825. (D) Expression levels of the overlapping genes identified in (C) within the GSE136825 dataset.

Analysis identified 2609 DEGs between the CRS and control groups (Fig. 4A). Subsequently, a heatmap was generated to depict the top 50 DEGs (Fig. 4B). By intersecting DEGs from CRS patients with the 19 overlapping targets, we aimed to further reveal potential molecular targets for GA in the treatment of CRS (Fig. 4C). A total of six key target genes were identified. Among them, ALOX5, HCK, LCN2, Syk, and TP53 were upregulated, while MPO was downregulated (Fig. 4D).

### 3.5 PPI network construction and analysis

**Fig. 5:**
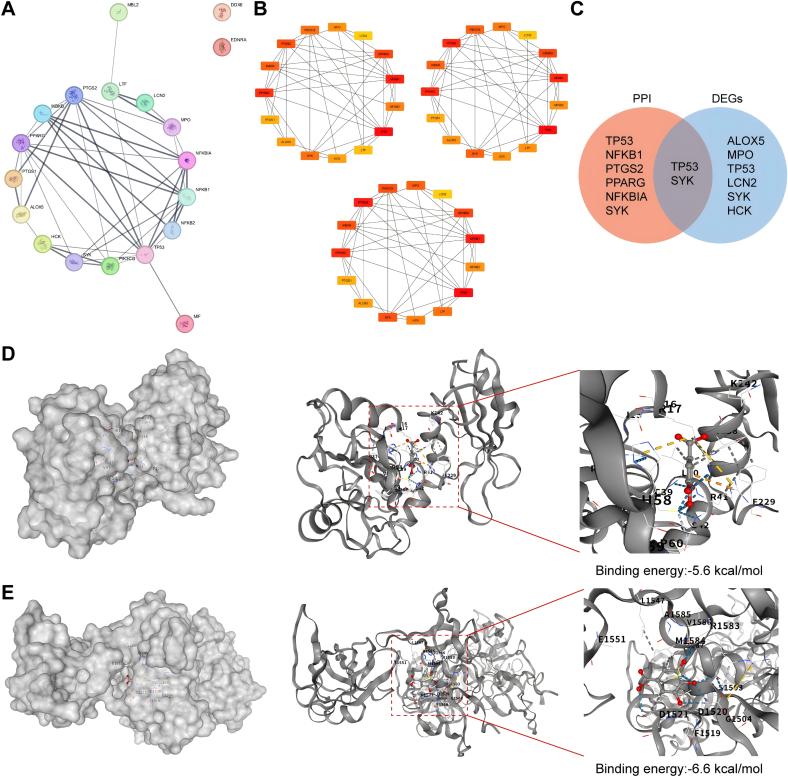
Protein-protein interaction network and molecular docking validation. (A) PPI network constructed from the common targets of GA and CRS. (B) Hub genes in the PPI network identified by three topological algorithms (MCC, Degree, and Closeness Centrality). (C) Venn diagram of the overlapping genes between the hub genes and the DEGs from GEO. (D-E) Molecular docking models showing the binding poses and interactions between GA and the potential target proteins (D) Syk and (E) TP53.

A PPI network comprising 19 nodes and 92 edges was constructed based on these 19 overlapping targets (Fig. 5A-B). To detect the pivotal proteins within the PPI network, the cytoHubba plugin was employed. Three ranking algorithms including MCC, Degree, and Closeness were applied. The top 6 candidate hub proteins identified by both the three algorithms were: TP53, NFKB1, PTGS2, PPARG, NFKBIA, and Syk. Subsequently, we overlapped the six key target proteins with the six previously identified hub DEGs. The intersection thereby yielded two common genes including TP53 and Syk (Fig. 5C).

### 3.6 Molecular docking analysis

Molecular docking was conducted to evaluate the potential interaction between GA and the identified target proteins. The binding affinity (expressed in kcal/mol) was calculated using CB-Dock2. Based on established practices in network pharmacology-based virtual screening, a binding energy below −5.0 kcal/mol was considered indicative of potential binding activity (Yao et al., 2025). Our findings indicate that GA exhibits favorable binding to the two key proteins, TP53 and Syk, as shown by the molecular docking results in Fig. 5D-E. The binding energies for both were below −5 kcal/mol. Furthermore, hydrogen-bond networks stabilize every ligand–macromolecule complex, supporting the predicted interaction.

### 3.7 Therapeutic effects of CP-GA-PKP on CRS mice

**Fig. 6:**
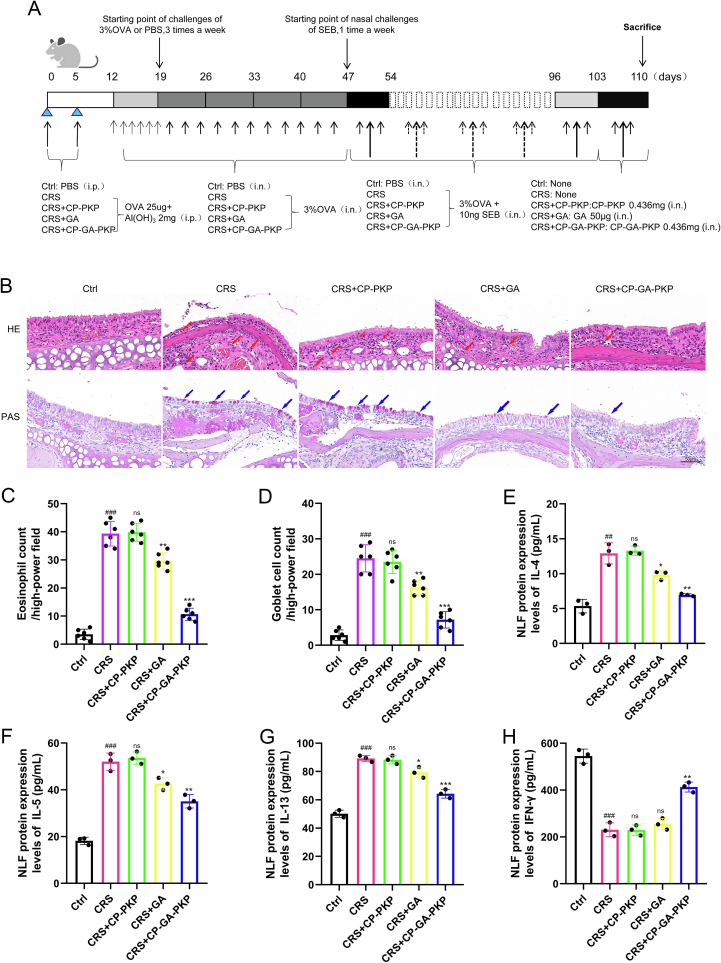
Evaluation of the CRS mouse model and therapeutic effects. (A) Schematic timeline of the experimental procedure. (B) Representative images of H&E and PAS staining of nasal mucosa from each group (×400). Red arrows indicate eosinophils; blue arrows indicate goblet cells. (C) Eosinophil count in the nasal mucosa. (D) Goblet cells count in the nasal mucosa. (*E*-H) Levels of cytokines in nasal lavage fluid were measured by ELISA. Data are presented as mean ± SD. ###*P* < 0.001 vs. the Ctrl group; *P < 0.05, **P < 0.01, ***P < 0.001 vs. the CRS group. (For interpretation of the references to colour in this figure legend, the reader is referred to the web version of this article.)

First, a CRS mouse model was established, followed by grouping for therapeutic intervention (Fig. 6A). The results demonstrated that treatment with free GA effectively ameliorated key histopathological features of CRS, namely eosinophil infiltration and goblet cell hyperplasia. Conversely, the empty nanocarrier (CP-PKP) showed no significant therapeutic effect compared to the CRS group. Strikingly, CP-GA-PKP co-treatment produced a more pronounced alleviation of these pathologies than free GA alone (Fig. 6B-D). Consistent with the histological improvements, free GA markedly curbed NLF concentrations of inflammatory mediators (IL-4, IL-5, IL-13, and IFN-γ), whereas CP-PKP remained inactive. This anti-inflammatory effect was further potentiated by CP-GA-PKP treatment (Fig. 6E-H).

**Fig. 7:**
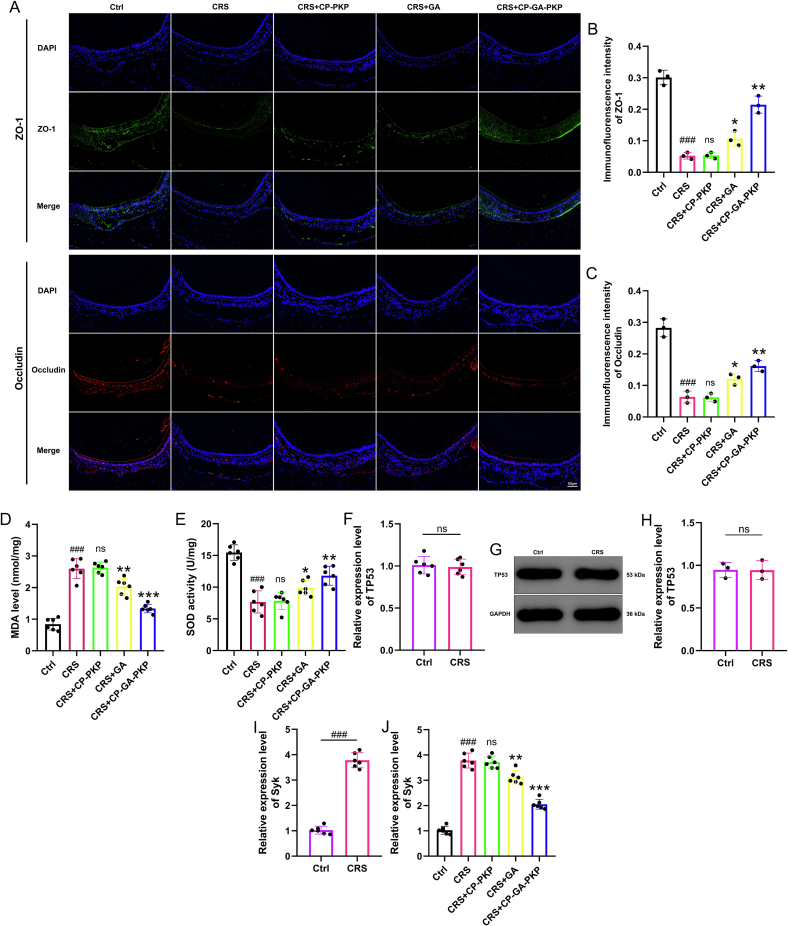
Intranasal CP-GA-PKP improves epithelial barrier function and alleviates oxidative stress in a CRS mouse model. (A) Representative immunofluorescence staining of ZO-1 and Occludin in the nasal mucosa. (B) Quantitative analysis of ZO-1 fluorescence intensity. (C) Quantitative analysis of Occludin fluorescence intensity. (D) Malondialdehyde (MDA) levels in the nasal mucosa. (E) Superoxide dismutase (SOD) activity in the nasal mucosa. (F) Relative mRNA expression of TP53. (G) Representative Western blot bands of TP53. (H) Relative protein expression levels of TP53 in control and CRS groups. (I-J) Relative mRNA expression of Syk. Data are presented as mean ± SD. ###*P* < 0.001 vs. the Ctrl group; ns, not significant, *P < 0.05, **P < 0.01, ***P < 0.001 vs. the CRS group.

At the molecular level, free GA administration up-regulated the epithelial junction proteins ZO-1 and Occludin, whereas CP-PKP alone produced no appreciable increase in these tight-junction components. However, the CP-GA-PKP most robustly enhanced their expression, suggesting its superior role in restoring mucosal barrier integrity (Fig. 7A-C). Furthermore, compared to controls, CRS nasal mucosa showed markedly elevated MDA content alongside a corresponding decrease in SOD activity, indicating enhanced oxidative stress. This trend was reversed upon free GA intervention, characterized by reduced MDA and elevated SOD levels. Treatment with CP-PKP did not significantly alter these oxidative stress markers. Notably, the reversal effect was most pronounced in the CP-GA-PKP group, indicating that the complete nanoformulation can most effectively alleviate CRS-induced oxidative stress (Fig. 7D-E).

### 3.8 Validation of key targets in CRS mice

The expression of TP53 and Syk in the nasal mucosa was investigated at the mRNA level. In addition, TP53 protein expression was assessed in the control and CRS groups. qRT-PCR analysis revealed that Syk gene expression was markedly elevated in the nasal mucosa of CRS mice and exhibited significant differences among all five experimental groups, whereas TP53 mRNA levels remained comparable to controls (Fig. 7F-J). TP53 protein expression showed no significant difference between the control and CRS groups. Furthermore, GA treatment significantly downregulated Syk mRNA levels in the nasal mucosa, an effect that was further enhanced by CP-GA-PKP. These results confirm that Syk, but not TP53, is dysregulated at the transcriptional level in this CRS model and responds to GA treatment. This supports the selection of Syk as the primary research target for subsequent mechanistic studies.

### 3.9 Safety evaluation of nano-drug

**Fig. 8:**
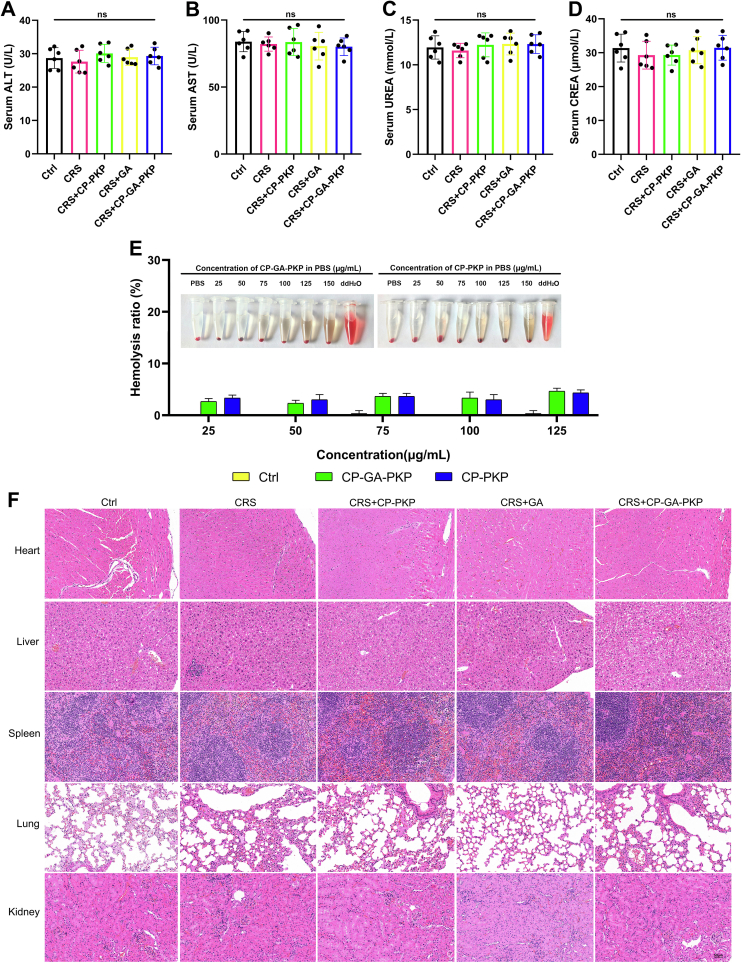
Biosafety evaluation. (A-D) Serum biochemical indices in C57BL/6 mice: (A) alanine aminotransferase (ALT), (B) aspartate aminotransferase (AST), (C) blood urea nitrogen (BUN), and (D) creatinine (CREA). (E) Hemolytic activity of CP-GA-PKP and CP-PKP at various concentrations. (F) Representative H&E-stained images of major organs (heart, liver, spleen, lung, and kidney) showing histological morphology. Data are presented as mean ± SD. ns, not significant.

To systematically assess the biological safety of the materials, we examined their impact on hepatic and renal function, hemolytic potential, and vital organs through a multidimensional experimental framework. Initially, serum biochemical parameters were quantified in mice 24 h post-intranasal dosing with an automated biochemical analyzer. Levels of ALT, AST, UREA, and CREA (Fig. 8A-D) did not differ significantly from control values, demonstrating that intranasal administration of these materials does not compromise liver or kidney function. To assess potential hematological risks following nasal administration, hemolysis assays were performed. The results confirmed that neither material induced notable hemolysis even at elevated concentrations (Fig. 8E), thereby corroborating their favorable hemocompatibility. Throughout the study, all experimental animals maintained normal behavioral patterns without exhibiting any adverse signs. H&*E*-stained sections displayed neatly arranged cardiac muscle fibers, preserved liver architecture, a clearly defined spleen cortex–medulla boundary, normal alveolar morphology, and an absence of pathological alterations in either glomeruli or renal tubules (Fig. 8F).

### 3.10 CP-GA-PKP ameliorates epithelial barrier dysfunction potently by synergistically suppressing the Syk/NF-κB pathway

**Fig. 9:**
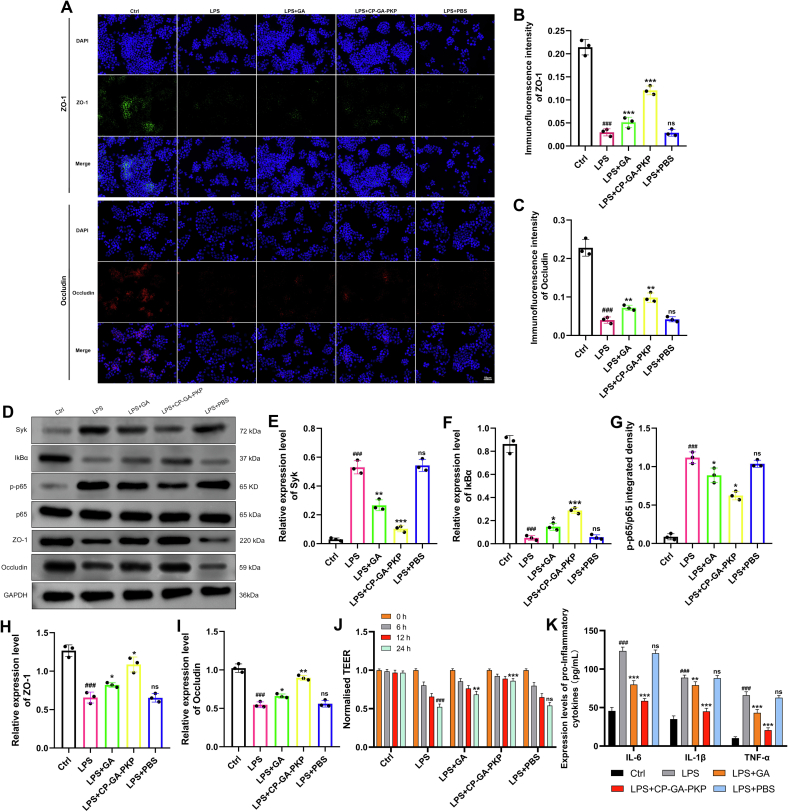
CP-GA-PKP alleviates inflammation and restores barrier function in HNEpCs via the Syk/NF-κB pathway. (A) Representative immunofluorescence images of ZO-1 and Occludin. (B—C) Quantitative analysis of ZO-1 and Occludin fluorescence intensity. (D) Representative Western blot bands of Syk, IκBα, p-p65, ZO-1, and Occludin. (E-I) Relative protein expression levels quantified from (D): (E) Syk, (F) IκBα, (G) p-p65/p65, (H) ZO-1, (I) Occludin. (J) TEER values of HNEpC monolayers measured at 0, 6, 12, and 24 h post-treatment. (K) Secreted cytokine levels (IL-6, IL-1β, and TNF-α) in the cell culture supernatant. Data are presented as mean ± SD. ###*P* < 0.001 vs. the Ctrl group; ns, not significant, *P < 0.05, **P < 0.01, ***P < 0.001 vs. the CRS group.

Immunofluorescence staining first showed that LPS stimulation lowered Occludin and ZO-1 levels in HNEpCs, while GA treatment restored them. This restoration was further enhanced by CP-GA-PKP treatment (Fig. 9A-C). Western blots corroborated the LPS-induced drop and GA-mediated rescue of Occludin and ZO-1, with CP-GA-PKP yielding the strongest rebound. Furthermore, LPS stimulation up-regulated Syk and phosphorylated p65, both of which were blocked by GA, with CP-GA-PKP exhibiting a stronger inhibitory effect (Fig. 9D-I). Furthermore, LPS stimulation induced degradation of IκBα, which was significantly attenuated by GA treatment. Consistent with the p65 results, CP-GA-PKP more potently preserved total IκBα protein levels compared to free GA. To functionally validate the epithelial barrier restoration suggested by ZO-1 and Occludin upregulation, we measured TEER in HNEpC monolayers. LPS stimulation caused a time-dependent decrease in TEER, indicating barrier disruption. Treatment with GA significantly attenuated this decline, and CP-GA-PKP further preserved TEER values, maintaining significantly higher barrier integrity compared to free GA at 24 h post-LPS stimulation (Fig. 9J). In parallel, LPS-elicited elevations of IL-1β, IL-6, and TNF-α responses in HNEpCs underwent suppression upon GA treatment; compared to GA alone, a more pronounced suppression was observed with CP-GA-PKP (Fig. 9K).

## 4 Discussion

CRS remains a challenging inflammatory disease characterized by persistent mucosal inflammation, epithelial barrier disruption, and tissue remodeling (Fokkens et al., 2020). Its complex pathophysiology involves multiple interconnected pathways, including oxidative stress, sustained inflammatory signaling, and impaired mucosal defense mechanisms (Orlandi et al., 2016). To address this complexity, and guided by network pharmacology predictions that identified Syk as a pivotal upstream target in CRS-related inflammatory networks, we developed a novel nanocomposite, CP-GA-PKP, which integrates targeted drug delivery with specific anti-inflammatory action. This platform demonstrated remarkably superior efficacy compared to free GA, which provided only moderate benefits. This enhanced performance is mediated through sophisticated mechanisms, primarily involving precise Syk/NF-κB pathway inhibition and epithelial-barrier restoration. In the nasal mucosa, the nanocomposite markedly reduces eosinophil infiltration and goblet cell hyperplasia, while also lowering levels of inflammatory factors in the NLF.

The differential therapeutic outcomes observed across experimental groups provide compelling evidence for the advantage of nanoformulation. Although GA possesses documented anti-oxidant and immunomodulatory activities (Kahkeshani et al., 2019; Ahn et al., 2016), its therapeutic potential is severely limited by pharmacokinetic challenges, including poor bioavailability and non-specific distribution (Yap et al., 2021). In marked contrast, the CRS + CP-GA-PKP group demonstrated significantly enhanced therapeutic outcomes across all evaluated parameters. This enhanced performance is attributed to the ROS-responsive drug release mechanism at the core of the nanodelivery system's design. This mechanism, based on thioketal bond cleavage, is intended to achieve targeted GA delivery specifically to inflamed tissues (Pan et al., 2020), a concept supported by our in vitro release data demonstrating rapid drug release under oxidative conditions (Fig. 2E). Furthermore, quantitative WST-1 assay confirmed that the Ce-MOF-Pt core possesses intrinsic SOD-like activity with an IC50 of 45.3 ± 4.5 μg/mL (Fig. 2F), providing direct biochemical evidence for its ROS-scavenging capacity. The pathological overproduction of reactive oxygen species in CRS tissues creates an ideal microenvironment for triggered drug release, resulting in higher local drug concentrations precisely at disease sites (Tai et al., 2023). This targeted delivery strategy represents a significant advancement over conventional administration methods, effectively addressing the pharmaceutical limitations that constrain free GA efficacy. Importantly, direct biochemical evidence confirmed the potent antioxidant capacity of CP-GA-PKP. MDA and SOD served as biomarkers of oxidative stress and antioxidant defense, respectively. Compared with the Ctrl group, CRS mice displayed marked oxidative damage, with MDA increased and SOD diminished. CP-GA-PKP reversed the imbalance by reducing MDA content and restoring SOD activity. This result demonstrates that CP-GA-PKP not only responds to ROS but also actively scavenges excess ROS, thereby ameliorating the oxidative microenvironment that perpetuates inflammation and barrier damage in CRS.

Given the relevance of Syk, a crucial regulator of immune cell activation and inflammatory signaling (Mócsai et al., 2010), in airway inflammation where it contributes to disease persistence (Geahlen, 2014), we first prioritized it as a potential key molecular target of GA through network pharmacology. Building upon this prediction, we experimentally validated that CP-GA-PKP treatment effectively suppresses Syk/NF-κB signaling. NF-κB orchestrates CRS inflammation, dictating transcription of multiple pro-inflammatory cytokines and chemokines (Wang et al., 2019). Our results showed that CP-GA-PKP markedly suppressed this pathway, as evidenced by reduced phosphorylation of p65 and, importantly, by attenuated degradation of its upstream inhibitor IκBα. These findings further confirm that GA interferes with the canonical NF-κB activation cascade, thereby inhibiting its downstream key drivers of CRS pathology (Tomassen et al., 2016). The enhanced inhibitory effect of the nanocomposite compared to free GA highlights the advantage of targeted delivery in improving intracellular drug bioavailability and efficacy at critical signaling nodes. A particularly significant finding was the robust upregulation of the tight-junction markers following CP-GA-PKP treatment. Epithelial barrier integrity, maintained by tight junction complexes, is crucial for nasal mucosal homeostasis and represents a key determinant of disease severity in CRS (Yan et al., 2024). Barrier dysfunction facilitates increased penetration of pathogens and allergens, perpetuating inflammatory responses and contributing to disease chronicity (Hammad and Lambrecht, 2015).

An important consideration arising from our data is the relationship between the predicted binding affinity of GA for Syk and the observed potent inhibition of this pathway. The molecular docking analysis indicated a moderate binding energy (−5.8 kcal/mol) between GA and Syk (Fig. 5D), which alone might not fully account for the robust suppression of Syk and downstream signaling. However, several lines of evidence suggest a more complex mechanism. First, our in vivo data revealed that Syk mRNA expression was significantly upregulated in the nasal mucosa of CRS mice (Fig. 7I), indicating transcriptional activation under inflammatory conditions. The observed reduction in Syk protein levels following CP-GA-PKP treatment (Fig. 7J) implies that GA may exert its effects, at least in part, by modulating Syk expression rather than solely through direct competitive inhibition. As a potent antioxidant, GA could influence redox-sensitive transcription factors that drive Syk gene expression. Second, the multi-target nature of GA—consistent with network pharmacology principles—means that its therapeutic effect likely results from synergistic actions on multiple nodes within the inflammatory network, including other targets identified in our screening (e.g., NFKBIA, PTGS2). The cumulative impact of modulating these interconnected targets can lead to significant pathway suppression, even if the direct interaction with any single node is moderate. Finally, the ROS-responsive release mechanism of CP-GA-PKP ensures high local concentration of GA precisely at sites of inflammation (Fig. 2E), enhancing bioavailability to compensate for moderate binding affinity. Thus, the robust inhibition of the Syk/NF-κB pathway observed with CP-GA-PKP reflects a combination of transcriptional regulation, multi-target synergy, and targeted delivery.

Furthermore, the relationship between the two core mechanisms of CP-GA-PKP—ROS scavenging and Syk/NF-κB inhibition—warrants further consideration. These mechanisms are not likely to be independent; rather, they are intricately linked in a positive feedback loop that perpetuates CRS pathology. Excessive ROS production in the inflamed sinonasal microenvironment can directly activate redox-sensitive signaling pathways (Finkel, 2011). Activation of this pathway drives the expression of pro-inflammatory cytokines and chemokines, which in turn recruit and activate more inflammatory cells (e.g., neutrophils, eosinophils), leading to further ROS generation (Vorobjeva et al., 2023). By potently scavenging ROS through its Ce-MOF-Pt nanozyme core and PDA shell, CP-GA-PKP disrupts this cycle at its inception, reducing the oxidative stimulus that activates Syk. Concurrently, the released GA directly suppresses Syk and downstream NF-κB signaling, thereby diminishing the inflammatory response and subsequent ROS production from inflammatory cells. Thus, we propose that the two mechanisms operate synergistically: ROS scavenging alleviates the upstream trigger of Syk activation, while Syk/NF-κB inhibition reduces the downstream source of ROS. This integrated, self-amplifying therapeutic effect distinguishes CP-GA-PKP from agents targeting a single pathway. A detailed dissection of the causal relationship between ROS dynamics and Syk represents an important direction for our future mechanistic studies.

The restoration of epithelial barrier integrity by CP-GA-PKP is a direct downstream consequence of its coordinated suppression of inflammatory signaling. Specifically, blocking Syk/NF-κB signaling decreases the production of cytokines, which are known to directly downregulate tight junction expression (Peng et al., 2022; He et al., 2012). The comprehensive action of CP-GA-PKP on both inflammatory processes and barrier repair mechanisms represents a significant advancement over conventional single-mechanism approaches. The marked performance difference between free GA and the nanocomposite formulation carries important implications for therapeutic development in CRS. The enhanced efficacy of CP-GA-PKP demonstrates that advanced drug delivery systems are essential components that can dramatically improve therapeutic outcomes. This is particularly relevant for natural compounds like GA, which often possess excellent biological activity but suffer from suboptimal pharmacokinetic properties (Kumar et al., 2024).

From a clinical perspective, our findings position Syk inhibition as a promising therapeutic strategy for CRS. While Syk inhibitors have shown potential in other inflammatory conditions (Masuda and Schmitz, 2008), their application in CRS represents a novel approach that targets upstream signaling events in the inflammatory cascade. The selection of Syk, initially prioritized through network pharmacology analysis, was thus mechanistically validated in our experimental models. The use of GA as a Syk inhibitor is particularly advantageous given its natural origin and multi-targeted actions (Bai et al., 2021). Furthermore, the ROS-responsive delivery system addresses the challenge of targeted therapy in the complex sinus anatomy, potentially offering improved efficacy with reduced systemic exposure. The barrier-restorative effects of CP-GA-PKP deserve special emphasis, as current CRS therapies often focus primarily on anti-inflammatory effects while neglecting epithelial repair. The demonstrated upregulation of Occludin and ZO-1 suggests that our therapeutic approach not only suppresses inflammation but also promotes structural and functional recovery of the nasal mucosa. This interpretation is further supported by our functional TEER assays, which showed that CP-GA-PKP treatment significantly preserved epithelial barrier integrity in LPS-challenged HNEpC monolayers. The concordance between increased tight junction protein expression and enhanced barrier function provides robust evidence for the barrier-restorative capacity of our nanozyme platform.

This comprehensive action represents a significant advancement in CRS management strategy. Notably, while network pharmacology also highlighted TP53 as a hub gene, our in vivo data did not show significant changes in its expression at the mRNA level in the CRS model. This suggests that the therapeutic effect of GA/CP-GA-PKP in this specific model may be more directly mediated through the Syk/NF-κB axis, or that TP53 involvement occurs under different pathological conditions or at the protein activity level.

Having established the therapeutic efficacy of CP-GA-PKP, it was equally critical to evaluate its biosafety profile, a fundamental prerequisite for any potential therapeutic agent, especially given that nasally administered agents undergo systemic absorption and their metabolites are primarily cleared renally, with potential off-target effects (Shehata et al., 2023; Ruan et al., 2024; Zhang et al., 2023; Katarey and Verma, 2016). To address this, CP-GA-PKP was comprehensively evaluated for effects on key organs, hematological parameters, and hemolytic potential. Reassuringly, all evaluated safety indices fell within normal physiological ranges, indicating no significant systemic toxicity and confirming a favorable biosafety profile for our nanocomposite.

The CP-GA-PKP platform offers distinct advantages over existing therapeutic strategies for CRS. Compared to conventional nanocarriers such as liposomes or polymeric micelles, which primarily function as passive drug delivery vehicles, our system features a therapeutically active core. The Ce-MOF-Pt nanozyme itself possesses intrinsic catalase and SOD-mimicking activities, enabling it to actively scavenge pathological ROS while simultaneously serving as a drug depot. This “nanocatalyst” approach transforms the drug delivery system from an inert carrier into an active participant in therapy. Furthermore, the ROS-responsive thioketal linker provides a more disease-specific release mechanism than pH-sensitive linkages, as the CRS microenvironment is characterized by elevated ROS rather than acidic pH (Tsai et al., 2025).

In comparison to synthetic Syk inhibitors (e.g., fostamatinib), GA offers a multi-targeted, network pharmacology-based mechanism derived from a natural product with an established safety profile. Our biosafety evaluation confirmed no significant hemolysis, hepatorenal toxicity, or organ damage, contrasting with concerns regarding immunosuppression or off-target effects associated with long-term use of synthetic kinase inhibitors (Fig. 8). Moreover, while existing Syk inhibitors focus primarily on anti-inflammation, CP-GA-PKP uniquely combines Syk/NF-κB inhibition with epithelial barrier restoration via GA-mediated upregulation of tight junction proteins. This dual action addresses both inflammatory and structural pathologies, representing a more holistic therapeutic approach.

Despite these promising findings, several limitations should be acknowledged. First, we did not directly visualize in vivo biodistribution of the nanozyme; techniques such as fluorescence labeling would provide more definitive evidence for targeted delivery and pharmacokinetic behavior. Second, comprehensive pharmacokinetic data (half-life, clearance pathways, organ accumulation) and maximum tolerated dose (MTD) are lacking—essential prerequisites for clinical translation. Third, the causal relationship between ROS scavenging and Syk/NF-κB inhibition requires further mechanistic dissection. Fourth, our efficacy evaluation was limited to a single eosinophilic CRS model (OVA/SEB-induced) with 1-week treatment duration, without dose-response studies or assessment of long-term efficacy and recurrence. Fifth, while our data strongly support Syk as a key functional target, genetic rescue experiments (e.g., Syk knockdown) would provide definitive proof of causality. Sixth, biosafety assessment was limited to short-term observation; long-term toxicity and immunogenicity studies are needed. Seventh, while we have quantitatively characterized the SOD-like activity of the Ce-MOF-Pt core, comprehensive enzyme kinetic characterization of its CAT-like activity and direct measurement of cellular ROS scavenging rate (e.g., via DCFH-DA assay) were not performed in this proof-of-concept study. These experiments would provide a more complete profile of the nanozyme's antioxidant capacity and are important directions for our future mechanistic investigations.

Future studies will address these limitations by: (1) performing in vivo imaging studies to track the biodistribution, metabolism, and clearance of fluorescently labeled CP-GA-PKP; (2) conducting dose-escalation studies to establish the MTD and therapeutic window; (3) designing mechanistic experiments to delineate the causal links between ROS scavenging and Syk pathway inhibition; (4) evaluating therapeutic efficacy in additional CRS models representing different endotypes; and (5) extending the safety assessment to chronic exposure scenarios; and (6) conducting comprehensive enzyme kinetic characterization of the nanozyme's CAT-like activity and cellular ROS scavenging capacity to fully elucidate its antioxidant mechanisms.

## 5 Conclusion

Our study demonstrates that the CP-GA-PKP nanocomposite achieves superior therapeutic outcomes in CRS through integrated mechanisms that combine targeted drug delivery with specific pharmacological actions. The superior efficacy of the complete nanoformulation, in stark contrast to the limited benefits of free GA alone, underscores the necessity of the integrated design. The ROS-responsive drug release ensures targeted delivery, which in turn enables the strong blockade of the Syk/NF-κB pathway and effective restoration of the epithelial barrier, collectively achieving comprehensive control of CRS pathology. These findings establish a promising nanotherapeutic platform for CRS in preclinical settings and firmly validate Syk inhibition as a viable therapeutic strategy. Moreover, the successful integration of targeted drug delivery with a mechanism-based therapeutic action establishes a new paradigm that may pave the way for the treatment of other airway inflammatory disorders, pending further pharmacokinetic and toxicological validation.

## CRediT authorship contribution statement

**Fangwei Zhou:** Writing – original draft, Methodology, Investigation, Data curation, Conceptualization. **Shanhu Gao:** Writing – original draft, Investigation, Conceptualization. **Tuotuo Xiong:** Methodology, Investigation. **Danyi Luo:** Software, Investigation. **Peixing Lin:** Software, Project administration. **Junbo Su:** Writing – review & editing, Supervision, Conceptualization. **Houyong Kang:** Writing – review & editing, Supervision, Project administration, Funding acquisition, Conceptualization.

## Declaration of competing interest

The authors declare no competing financial interests or personal relationships that could have appeared to influence the work reported in this paper.

## Data Availability

Data will be made available on request.
